# Prevalence of Concurrent Functional Vision and Hearing Impairment and Association With Dementia in Community-Dwelling Medicare Beneficiaries

**DOI:** 10.1001/jamanetworkopen.2021.1558

**Published:** 2021-03-19

**Authors:** Pei-Lun Kuo, Alison R. Huang, Joshua R. Ehrlich, Judith Kasper, Frank R. Lin, Michael M. McKee, Nicholas S. Reed, Bonnielin K. Swenor, Jennifer A. Deal

**Affiliations:** 1 Department of Epidemiology, Johns Hopkins Bloomberg School of Public Health, Baltimore, Maryland; 2 Cochlear Center for Hearing and Public Health, Johns Hopkins Bloomberg School of Public Health, Baltimore, Maryland; 3 Department of Mental Health, Johns Hopkins Bloomberg School of Public Health, Baltimore, Maryland; 4 Department of Ophthalmology and Visual Sciences, University of Michigan Medical School, Ann Arbor; 5 Institute for Healthcare Policy and Innovation, University of Michigan, Ann Arbor; 6 Department of Health Policy and Management, Johns Hopkins Bloomberg School of Public Health, Baltimore, Maryland; 7 Department of Otolaryngology–Head and Neck Surgery, Johns Hopkins School of Medicine, Baltimore, Maryland; 8 Department of Family Medicine, University of Michigan Medical School, Ann Arbor; 9 Disability Health Research Center, Johns Hopkins Bloomberg University, Baltimore, Maryland; 10 The Wilmer Eye Institute, Johns Hopkins University, Baltimore, Maryland

## Abstract

**Importance:**

Impairments in vision or hearing are common and have been independently linked to higher risk of dementia in older adults. There is a limited understanding of the prevalence of concurrent functional vision and hearing impairment (dual sensory impairment) and its contribution to dementia risk.

**Objective:**

To examine the age-specific prevalence of functional dual sensory impairment among older adults, and to investigate the cross-sectional and 7-year longitudinal associations between functional dual sensory impairment and dementia.

**Design, Setting, and Participants:**

This cohort study of 7562 older adults used data from the US National Health and Aging Trends Study (NHATS), a nationally representative cohort study of community-dwelling, Medicare beneficiaries aged 65 years and older in the US. Participants in the study with complete data on hearing, vision, and dementia were included in analysis. Data were collected between 2011 and 2018, and between March 2018 and May 2020.

**Exposures:**

Self-reported functional sensory impairments (ie, no sensory impairment, functional vision impairment only, functional hearing impairment only, and functional dual sensory impairment).

**Main Outcomes and Measures:**

Age-specific prevalence of functional sensory impairments was calculated. Generalized linear regression with a complementary log-log link and a discrete time proportional hazards model with a complementary log-log link were used to assess the cross-sectional and 7-year longitudinal hazard of dementia.

**Results:**

Of 7562 participants, 3073 (40.7%) were ages 80 years or older and 4411 (58.3%) were women. Overall, 5.4% (95% CI, 4.7%-6.1%) of participants reported functional vision impairment only, 18.9% (95% CI, 18.9%-17.8%) reported functional hearing impairment only, and 3.1% (95% CI, 2.7%-3.5%) reported functional dual sensory impairment (prevalence estimates are weighted). Participants reporting sensory impairments were older (no impairment: age ≥90 years, 2.12% [95% CI, 1.79%-2.46%] vs functional dual sensory impairment: age ≥90 years, 20.06% [95% CI, 16.02%-24.10%]), had lower education (no impairment: <high school, 19.05% [95% CI, 17.27%-20.83%] vs functional dual sensory impairment: <high school, 46.15% [95% CI, 38.38%-53.92%]), and greater disease burden (eg, heart disease: no impairment, 15.30% [95% CI, 14.04%-16.55%] vs functional dual sensory impairment, 25.49% [95% CI, 19.96%-31.02%]). Compared with no impairment, functional vision impairment (adjusted hazard ratio [aHR], 1.89; 95% CI, 1.57-2.28), functional hearing impairment (aHR, 1.14; 95% CI, 1.00-1.31), and functional dual sensory impairment (aHR, 2.00; 95% CI, 1.57-2.53) were associated with a higher cross-sectional hazard of dementia. Over 7 years, functional vision impairment (aHR, 1.40; 95% CI, 1.12-1.74), functional hearing impairment (aHR, 1.09; 95% CI, 0.95-1.24), and functional dual sensory impairment (aHR, 1.50; 95% CI, 1.12-2.02) were associated with a higher hazard of incident dementia compared with no impairment.

**Conclusions and Relevance:**

In this cohort study of US Medicare beneficiaries, dual sensory impairment was prevalent in older adults and associated with increased risk of dementia. These findings suggest that sensory rehabilitative interventions for multiple impairments may be an additional resource in efforts to reduce dementia risk.

## Key Points

**Question:**

What is the prevalence of functional dual sensory impairment and is it associated with dementia risk among community-dwelling older adults in the US?

**Findings:**

In this cohort study of adults over the age of 65 years, 14.9% of adults aged 90 years or older reported functional dual sensory impairment. Functional dual sensory impairment was associated with a higher 7-year risk of dementia compared with no sensory impairment.

**Meaning:**

The results of this study suggest that functional sensory impairments are prevalent among older adults, and interventions to address multiple sensory impairments may be a potential target to reduce dementia risk.

## Teaser

This cohort study of community-dwelling older adults in the US examined the age-specific prevalence of functional dual sensory impairment and assessed cross-sectional and longitudinal associations between functional dual impairment and dementia.

## Introduction

In the US, over 5.8 million older adults have dementia. In the absence of effective prevention and treatment, the number of older US adults with dementia is expected to rise to 13.8 million by 2050.^1,2^ Reducing dementia risk through identification of and intervention on potentially modifiable risk factors is a public health priority.^3^

Vision and hearing impairments have been increasingly recognized as potentially modifiable risk factors for dementia in older adults. Vision impairment has been shown to be associated with higher risk of dementia, with studies reporting up to an 8 times higher hazard of dementia among those with vision impairment.^4,5,6,7^ A 2020 meta-analysis conducted by the Lancet Commission on dementia prevention, intervention, and care^3^ also reported that, of 12 identified modifiable risk factors for dementia, hearing loss has the highest population attributable fraction for dementia risk. Hearing loss nearly doubles risk for dementia.^8^ Hearing and vision impairment may increase dementia risk through several mechanisms, such as changes in brain structure and function, increased cognitive load,^9,10^ depression,^11,12^ social isolation,^13,14,15,16^ and reduced physical activity.^17,18,19^

Prior work on sensory loss and dementia has primarily focused on dementia risk associated with vision or hearing impairment in isolation; however, co-occurrence of sensory impairments among older adults is common. More than 1 in 10 adults aged 80 years or older have concurrent hearing and vision impairment (ie, dual sensory impairment).^20^ Persons with dual sensory impairment may be a particularly vulnerable subgroup because, unlike individuals with single sensory impairment, it is more difficult to employ sensory substitution (eg, using the auditory system to compensate for loss in functioning associated with visual impairment or vice versa) to preserve functioning.

Thus, persons with dual sensory impairment may have particularly higher risk for dementia—yet few studies have investigated this association. Of the small number of existing studies, common limitations include use of highly selected populations (with reduced generalizability of study findings) and limitations in assessing longitudinal associations given cross-sectional study designs.^21,22,23^ There is also limited representation of older adults over 90 years in prior studies because study samples comprise mostly younger participants.^20,24^

Additionally, there is a lack of evidence describing the prevalence of functional hearing, vision, and dual sensory impairment and its association with dementia risk. Functional sensory impairment is a construct distinct from objectively measured sensory impairment that independently affects health.^25,26^ Functional sensory impairment captures the day-to-day impact of sensory impairment by incorporating self-perception, awareness, and compensation mechanisms. This important measure may provide a comprehensive assessment of the association of sensory impairment with dementia risk by measuring sensory impairment from the perspective of functional disability.

In this study, we use data from the National Health and Aging Trends Study (NHATS), a nationally representative study of Medicare beneficiaries in the US, to first examine the age-specific prevalence of functional sensory impairments among older adults. Second, we investigate the cross-sectional and longitudinal associations between functional sensory impairments (hearing, vision, and dual sensory impairment) and prevalent and incident dementia over 7 years of follow-up. We hypothesize that prevalence of dementia and risk of dementia over time will be greatest among participants with functional dual sensory impairment. This study is among the first to examine the association between functional sensory impairments and incident dementia in the US using a large, population-based sample.

## Methods

### Study Participants

NHATS, an ongoing nationally representative sample of Medicare beneficiaries aged 65 and older in the US, is a longitudinal study with annual follow-up.^27^ Medicare is a government program that provides health care to nearly all older adults 65 years and older and some younger individuals. This study uses 7-year longitudinal data collected between 2011 and 2018. At baseline, 8245 participants were sampled and annual interview data was collected by direct contact with either the participant or a proxy respondent. Excluded from our analyses were 468 participants who were nursing home residents and 168 persons living in other residential facilities from whom sensory impairment and dementia were not collected. Among the 7609 participants with information about dementia status, 47 participants who did not have complete self-reported vision and hearing information were excluded. Therefore, 7562 participants with complete hearing, vision, and dementia information at the baseline were included in our analysis. This study uses publicly available, nonidentifiable data and was approved by the John Hopkins University institutional review board. Informed consent was obtained by NHATS investigators from all participants or their proxy respondents. This study adheres to the Strengthening the Reporting of Observational Studies in Epidemiology (STROBE) reporting guideline.

### Measures

#### Dementia

Dementia status was determined using self-reported diagnosis information and measured cognitive performance in 3 domains (memory [immediate and delayed 10-word recall], orientation [date, month, year, and day of the week; naming the President and Vice President], and executive function [clock drawing test]).^28^ Participants were classified as having probable, possible, or no dementia. Participants classified with probable dementia self-reported or had a proxy respondent report a dementia diagnosis, had a proxy respondent provide a score on the AD8 Dementia Screening Interview indicating probable dementia, or scored at or below 1.5 SD from the mean of self-respondents in at least 2 of 3 measured cognitive domains. Participants with possible dementia scores at or below 1.5 SD from the mean in at least 1 cognitive domain. All other participants were classified as having no dementia.

The broader definition of dementia (defined as possible or probable dementia) is used in this study and has high sensitivity (85.7%) and specificity (83.7%) of dementia diagnosis compared with clinical diagnosis of dementia in the Aging, Demographics, and Memory Study.^28^ Dementia classification is available for all rounds of data collection.

#### Functional Sensory Impairment

Functional sensory impairment is an individual’s self-reported, perceived difficulty with vision and hearing in common situations in regular aided condition, if applicable. Functional vision and hearing impairment were assessed by a series of questions regarding difficulty seeing and/or hearing in certain situations. Participants are considered to have functional vision impairment if they reported blindness, an inability to see well enough to recognize someone across the street, or inability to see well enough to read newspaper print. Participants are considered to have functional hearing impairment if they reported deafness, hearing aid use, inability to hear well enough to use the telephone, or inability to hear well enough to carry on conversation in a room with the television or radio playing. Participants reporting both functional vision and hearing impairment were classified as having functional dual sensory impairment. For analysis, participants were categorized into 4 mutually exclusive groups (ie, no sensory impairment, functional vision impairment only, functional hearing impairment only, and functional dual sensory impairment) based on their reported sensory status at baseline.

### Covariates

Demographic characteristic covariates included sex, age (categorized into 6 age intervals [65-69, 70-74, 75-79, 70-84, 85-89, and ≥90+ years]), education (less than high school, high school, and greater than high school), and race/ethnicity (non-Hispanic White, non-Hispanic Black, Hispanic, and other). Diagnosis of diabetes, hypertension, stroke, heart attack, heart disease, lung disease, cancer, and history of smoking (ie, former or current) were assessed by self-report.

### Statistical Analysis

To address the age-specific prevalence of functional sensory impairments among older adults, we calculated the prevalence and distribution (ie, weighted proportion) of sensory impairment categories by age group. To investigate the cross-sectional and longitudinal associations between functional sensory impairments and prevalent and incident dementia, we began with a descriptive analysis. To compare baseline characteristics across the 4 sensory impairment categories, the weighted distribution of baseline covariates (age, sex, education, race/ethnicity, hypertension, diabetes, and smoking status) was calculated by sensory impairment status. Generalized linear regression with a complementary log-log link was used to model the cross-sectional hazard ratio of dementia by sensory impairment status at round 1. Data were fit to 3 models: unadjusted; adjusted for age, sex, education, and race/ethnicity; and adjusted for smoking status, hypertension, diabetes, stroke, heart attack, heart disease, lung disease, and cancer. Survey weights were used to account for the complex sampling survey design.

To investigate the longitudinal associations between functional sensory impairments and incident dementia, 4546 participants without dementia and with at least 1 follow-up visit were included in a discrete time survival analysis. Complementary log-log plots across 4 groups were used to assess the proportional hazard assumption. The proportional hazard assumption was checked, and time-varying hazard ratios were allowed when the assumption was violated. As with the cross-sectional analysis, we took a nested model building approach. We checked the proportional hazard assumptions by testing other interaction terms between time interval group indicators. Point estimates and 95% confidence intervals are reported.

Two sensitivity analyses were conducted. Out of concern for potential reverse confounding, where ratings of sensory impairment may be influenced by poor cognitive status, we imposed a 2-year lag in sensitivity analyses where assessment of dementia risk began 2 years after the measurement of sensory impairment. Participants who developed dementia within 2 years of measurement of sensory impairment were excluded, thus minimizing the risk of reverse confounding. In a separate sensitivity analysis, we restricted the sample to participants aged 65 to 85 years to reduce potential residual confounding by age. All analyses were conducted using SAS version 9.4 (SAS Institute) and R version 3.6 (R Project for Statistical Computing). Data were analyzed between March 2018 and May 2020.

## Results

Overall, 5.4% (95% CI, 4.7%-6.1%) of participants reported functional vision impairment only, 18.9% (95% CI, 18.9%-17.8%) reported functional hearing impairment only, 3.1% (95% CI, 2.7%-3.5%) reported functional dual sensory impairment, and 72.6% (95% CI, 71.3%-73.9%) reported no impairments (Table 1, Figure). In comparison with participants reporting no impairment, a higher proportion of participants reporting functional vision, hearing, or dual sensory impairment had less than a high school education (no impairment: <high school, 19.05% [95% CI, 17.27%-20.83%] vs functional dual sensory impairment: <high school, 46.15% [95% CI, 38.38%-53.92%]), diabetes (22.43% [95% CI, 21.10%-23.76%] vs 32.93% [95% CI, 27.24%-38.62%]), hypertension (62.98% [95% CI, 61.40%-64.55%] vs 71.27% [95% CI, 64.32%-78.22%]), stroke (8.11% [95% CI, 7.35%-8.87%] vs 19.91% [95% CI, 15.22%-24.61%]), heart attack (12.11% [95% CI, 11.02%-13.20%] vs 19.54% [95% CI, 14.41%-24.67%]), heart disease (15.30% [95% CI, 14.04%-16.55%] vs 25.49% [95% CI, 19.96%-31.02%]), and lung disease (14.30% [95% CI, 13.37%-15.23%] vs 18.74% [95% CI, 13.38%-24.10%]) (Table 2). Additionally, among Hispanic or non-Hispanic Black participants a higher proportion reported functional vision impairment and a lower proportion reported functional hearing impairment (eg, non-Hispanic Black participants, weighted percentage: vision impairment, 13.75% [95% CI, 11.51%-15.99%] vs no impairment, 8.90% [95% CI, 7.90%-9.90%]; hearing impairment, 3.76% [95% CI, 3.10%-4.42%]). Compared with individuals reporting no impairment, a higher proportion of participants reporting functional dual sensory impairment were Hispanic (weighted percentage: 17.39% [95% CI, 10.45%-24.32%] vs 6.44% [95% CI, 5.35%-7.52%]) and a lower proportion were non-Hispanic Black (5.95% [95% CI, 4.07%-7.83%] vs 8.90% [95% CI, 7.90%-9.90%]).

**Table 1.** Age-Specific Prevalence of Self-Reported Sensory Impairments^a^

Age group, y	No impairment, % (95% CI)^b^ (n = 5249)	Impairment only, % (95% CI)^b^		Dual sensory impairment, % (95% CI)^b^ (n = 303)
		Vision (n = 491)	Hearing (n = 1519)	
65-69	83.53 (81.48-85.57)	5.07 (3.95-6.19)	10.05 (8.24-11.86)	1.35 (0.71-1.99)
70-74	79.72 (77.42-82.02)	3.68 (2.43-4.92)	15.21 (12.98-17.44)	1.39 (0.73-2.04)
75-79	72.30 (69.70-74.91)	4.46 (3.29-5.63)	20.22 (17.78-22.66)	3.02 (2.17-3.86)
80-84	63.88 (60.67-67.08)	6.85 (5.46-8.24)	26.42 (23.56-29.27)	2.86 (1.96-3.76)
85-89	50.32 (46.84-53.80)	8.52 (6.69-10.35)	32.95 (29.69-36.21)	8.21 (6.85-9.57)
≥90	37.06 (33.23-40.90)	10.62 (8.03-13.22)	37.44 (33.52-41.37)	14.87 (11.90-17.84)

^a^ Sensory impairment categories are mutually exclusive.

^b^ Percentages weighted by age group.

**Figure.**
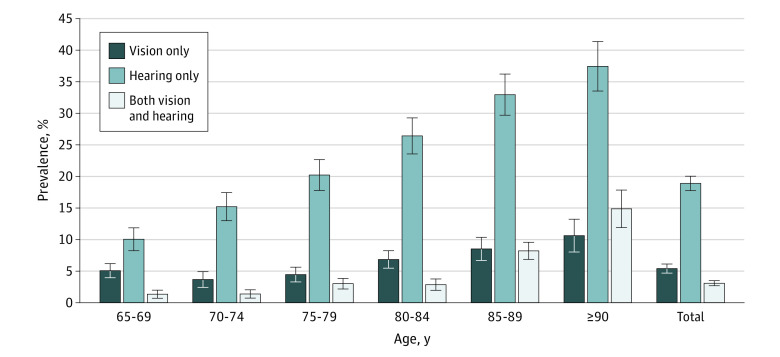
Prevalence of Older Adults by Functional Vision and Hearing Impairment Status: National Health and Aging Trends Study, 2011 Sensory impairment categories are mutually exclusive. Error bars indicate 95% CIs.

**Table 2.** Distribution of Baseline Characteristics Among Participants Stratified by Sensory Status^a^

Characteristics	No impairment, % (95% CI)	Impairment only, % (95% CI)		Dual sensory impairment, % (95% CI)
		Vision	Hearing	
Age group, y				
65-69	32.23 (31.08-33.39)	26.26 (21.96-30.57)	14.90 (12.52-17.29)	12.30 (7.15-17.44)
70-74	27.48 (26.45-28.52)	17.01 (12.45-21.58)	20.16 (17.65-22.66)	11.27 (6.13-16.40)
75-79	18.98 (17.91-20.04)	15.71 (12.06-19.36)	20.39 (17.84-22.95)	18.65 (13.88-23.42)
80-84	12.91 (12.07-13.75)	18.57 (15.04-22.10)	20.51 (18.46-22.57)	13.61 (9.38-17.85)
85-89	6.28 (5.62-6.94)	14.27 (10.85-17.70)	15.79 (13.96-17.63)	24.12 (20.56-27.68)
≥90	2.12 (1.79-2.46)	8.17 (6.24-10.10)	8.24 (6.97-9.51)	20.06 (16.02-24.1)
Sex				
Female	58.3 (56.54-60.06)	69.81 (64.75-74.86)	45.87 (43.46-48.27)	59.99 (53.96-66.03)
Male	41.7 (39.94-43.46)	30.19 (25.14-35.25)	54.13 (51.73-56.54)	40.01 (33.97-46.04)
Education				
<High school	19.05 (17.27-20.83)	33.30 (28.17-38.43)	23.11 (20.35-25.87)	46.15 (38.38-53.92)
High school	27.12 (25.48-28.75)	26.76 (23.06-30.46)	28.00 (25.93-30.08)	27.26 (20.96-33.57)
>High school	52.66 (50.31-55.00)	39.26 (33.74-44.78)	47.38 (44.50-50.25)	24.30 (18.56-30.04)
Race				
Non-Hispanic White	80.08 (78.25-81.90)	72.08 (67.32-76.84)	86.28 (84.21-88.35)	70.79 (63.60-77.98)
Hispanic	6.44 (5.35-7.52)	9.98 (6.13-13.83)	5.31 (4.07-6.55)	17.39 (10.45-24.32)
Non-Hispanic Black	8.90 (7.90-9.90)	13.75 (11.51-15.99)	3.76 (3.10-4.42)	5.95 (4.07-7.83)
Other^b^	4.58 (3.45-5.71)	4.19 (1.79-6.60)	4.65 (3.34-5.96)	5.87 (2.86-8.88)
Hypertension	62.98 (61.40-64.55)	72.16 (66.91-77.41)	63.69 (61.22-66.15)	71.27 (64.32-78.22)
Diabetes	22.43 (21.10-23.76)	32.82 (28.29-37.36)	25.14 (22.43-27.85)	32.93 (27.24-38.62)
Stroke	8.11 (7.35-8.87)	22.27 (18.52-26.01)	11.69 (9.86-13.51)	19.91 (15.22-24.61)
Heart attack	12.11 (11.02-13.20)	18.25 (14.45-22.05)	19.54 (14.42-24.67)	19.54 (14.41-24.67)
Heart disease	15.30 (14.04-16.55)	26.12 (21.56-30.66)	21.31 (19.04-23.57)	25.49 (19.96-31.02)
Lung disease	14.30 (13.37-15.23)	22.44 (16.90-27.98)	16.57 (14.52-18.61)	18.74 (13.38-24.10)
Cancer	24.70 (23.18-26.22)	23.32 (18.48-26.17)	30.87 (28.73-33.02)	24.01 (18.30-29.72)
Ever smoked	52.81 (51.01-54.60)	51.43 (45.72-57.15)	53.47 (50.22-56.72)	43.78 (35.48-52.07)
Dementia	16.57 (15.03-18.11)	39.58 (33.78-45.37)	25.76 (22.69-28.83)	55.99 (48.65-63.32)

^a^ Sensory impairment categories are mutually exclusive. Summation within each category may be slightly lower than 100% because a very small proportion of data are missing.

^b^ Other includes all respondents who did not report as non-Hispanic White, Hispanic, or non-Hispanic Black in the National Health and Aging Trends Study data set.

Prevalence of functional sensory impairments increased with older age (Table 1). The prevalence of functional vision impairment only was 5.1% (95% CI, 4.0%-6.2%) in the 65-to-69–years age group and decreased slightly in the 70-to-74–year (3.7% [95% CI, 2.4%-4.9%]) and 75-to-79–year (4.5% [95% CI, 3.3%-5.6%]) age groups. Prevalence of functional vision impairment only increased to 10.6% (95% CI, 8.0%-13.2%) in older adults over 90 years. The prevalence of functional hearing impairment only and functional dual sensory impairment increased with age. Prevalence of functional hearing impairment only was 10.1% (95% CI, 8.2%-11.9%) in the group aged 65 to 69 years and 37.4% (95% CI, 33.5%-41.4%) in the 90 years and older age group. Prevalence of functional dual sensory impairment was 1.4% (95% CI, 0.7%-2.0%) in the group aged 65 to 69 years and 14.9% (95% CI, 11.9%-17.8%) in the group aged 90 years or more.

In cross-sectional analysis, in comparison with older adults reporting no sensory impairment, functional vision impairment alone (adjusted hazard ratio [aHR], 1.89; 95% CI, 1.57-2.28), functional hearing impairment alone (aHR, 1.14; 95% CI, 1.00-1.31), and functional dual sensory impairment (aHR, 2.00; 95% CI, 1.57-2.53) were associated with a higher hazard of dementia after adjusting for age, sex, levels of education, race/ethnicity, disease burden, and smoking (model 3 in Table 3).

**Table 3.** Complementary Log-Log Regression Modeling the Cross-Sectional Association Between Sensory Impairment and Dementia

Status of sensory impairment	Hazard ratio (95% CI)		
	Model 1^a^	Model 2^b^	Model 3^c^
None	1 [Reference]	1 [Reference]	1 [Reference]
Vision only	2.83 (2.33-3.46)	2.01 (1.66-2.43)	1.89 (1.57-2.28)
Hearing only	1.65 (1.44-1.91)	1.17 (1.02-1.34)	1.14 (1.00-1.31)
Dual sensory impairment	4.57 (3.70-5.63)	2.10 (1.66-2.67)	2.00 (1.57-2.53)

^a^ Unadjusted model.

^b^ Adjusted for age, sex, levels of education, and race/ethnicity.

^c^ Adjusted for age, sex, levels of education, race/ethnicity, smoking history, hypertension, diabetes, stroke, heart attack, heart disease, lung disease, and cancer.

Over 7 years of follow-up (median [interquartile range] follow-up of 4 [2-7] years), in comparison with participants reporting no sensory impairment, functional vision impairment alone (aHR, 1.40; 95% CI, 1.12-1.74), functional hearing impairment alone (aHR, 1.09; 95% CI, 0.95-1.24), and functional dual sensory impairment (aHR, 1.50; 95% CI, 1.12-2.02) were associated with a higher hazard of incident dementia after adjusting for age, sex, levels of education, race/ethnicity, disease burden, and smoking (model 3 in Table 4).

**Table 4.** Discrete Time Survival Analysis Modeling the Association Between Sensory Impairment and Incident Dementia^a^

Status of sensory impairment	Hazard Ratio (95% CI)		
	Model 1^b^	Model 2^c^	Model 3^d^
None	1 [Reference]	1 [Reference]	1 [Reference]
Vision only	1.85 (1.49-2.29)	1.45 (1.16-1.80)	1.40 (1.12-1.74)
Hearing only	1.44 (1.27-1.63)	1.11 (0.97-1.26)	1.09 (0.95-1.24)
Dual sensory impairment	3.05 (2.29-4.06)	1.54 (1.14-2.07)	1.50 (1.12-2.02)

^a^ The proportional hazard assumption was checked, and time-varying hazard ratios were allowed when the assumption was violated. The proportional hazard assumption held for the functional hearing impairment group (vs no impairment) and for the functional vision impairment group (vs no impairment) across the 7-year follow-up period. For the functional dual sensory impairment group (vs no impairment), the proportional hazard assumption held across the first 4-year follow-up period, but did not hold after the 4-year follow-up. Time-varying hazard ratios were allowed between the dual sensory impairment group (vs no impairment) after 4-year follow-up.

^b^ Unadjusted model.

^c^ Adjusted for age, sex, levels of education, and race/ethnicity.

^d^ Adjusted for age, sex, levels of education, race/ethnicity, smoking history, hypertension, diabetes, stroke, heart attack, heart disease, lung disease, and cancer.

Estimates from the first sensitivity analysis, in which a 2-year lag in assessment of dementia risk was imposed, are in the same direction and are stronger in magnitude as estimates from the primary analysis without any change in inference when compared with no impairment (functional vision impairment alone: aHR, 1.42 [95% CI, 1.00-2.01]; functional hearing impairment alone: aHR, 1.29 [95% CI, 1.07-1.56]; functional dual sensory impairment: aHR, 2.15 [95% CI, 1.25-3.69]) (see eTable 1 in the Supplement). Results from the second sensitivity analysis, in which analysis was restricted to participants aged 65 to 85 years, are in the same direction compared with no impairment and, with the exception of hearing impairment, are stronger in magnitude as estimates from the primary analysis (functional vision impairment alone: aHR, 1.47 [95% CI, 1.14-2.91]; functional hearing impairment alone: aHR, 1.04 [95% CI, 0.89-1.23]; functional dual sensory impairment: aHR, 1.65 [95% CI, 1.07-2.52]) (see eTable 2 in the Supplement).

## Discussion

In a cohort of community-dwelling Medicare beneficiaries in the US, 5.4% of participants reported functional vision impairment only, 18.9% reported functional hearing impairment only, 3.1% reported functional dual sensory impairment, and 72.6% reported no impairments. Functional sensory impairment increased with age, with 1 in 7 older adults aged over 90 years reporting functional dual sensory impairment. Functional vision, hearing, and dual sensory impairment were also associated with 1.9-fold, 1.1-fold, 2.0-fold increases in the cross-sectional hazard of dementia, respectively, compared with those reporting no sensory impairment. Further, among participants without dementia at baseline, functional sensory impairment was associated with a higher likelihood of incident dementia over 7 years of follow-up. The strongest association was observed for participants who reported functional dual sensory impairment; compared with no impairment, functional dual sensory impairment was associated with a 50% higher hazard of incident dementia over 7 years.

This study is among the first, to our knowledge, to investigate the association between functional dual sensory impairment and incident dementia in a national sample of community-dwelling older adults in the US. This study adds to the limited data on functional sensory impairment, particularly functional dual sensory impairment, and dementia risk. Functional sensory impairment captures sensory impairment from the perspective of the individual, aligns with a disability framework, and provides insight on perceived impairment.^29^ Functional sensory impairment factors into overall health independent of objectively measured sensory impairment.^25,26^ For example, limitations in social and work activities are more strongly associated with functional vs objective hearing impairment.^25^ Self-reported vision impairment is also associated with risk of falls even after adjusting for objectively measured vision.^26^ Findings from studies of functional sensory impairment contribute to a more comprehensive understanding of how sensory impairment affects dementia risk by complementing studies that investigate objectively measured sensory impairment.

Prevalence of functional sensory impairments in the current study differs slightly from prevalence reported in other nationally representative studies that measure self-reported sensory impairment. In the Health and Retirement Study (HRS), 13.5% reported visual impairment, 7.8% reported hearing impairment, and 5.0% reported dual sensory impairments.^30^ Slight differences in prevalence of functional sensory impairment between the current study and HRS may be due to differences in functional sensory impairment measures. Sensory impairment measured in the HRS is measured by single-item measures asking participants to rate their hearing or eyesight on a scale from poor to excellent. Additionally, prevalence of functional sensory impairments is lower in NHATS compared with age-specific prevalence of objective sensory impairments reported in the National Health and Nutritional Examination Surveys (NHANES).^20^ Differences in method of measurement (ie, sensory impairment was measured objectively in NHANES) may account for differences in observed prevalence.

Despite increased interest in the sensory-cognitive relationship, there has been limited research examining the association between functional sensory impairments, particularly functional dual sensory impairment, and dementia. Our findings are consistent with existing prior studies.^23,24,31^ Notably, a longitudinal study of older US adults from the Health and Retirement Study^24^ observed a 26% higher hazard of dementia among participants who self-reported visual impairment compared with those with no visual impairment. This study also observed associations between hearing impairment (vs no hearing impairment) and dual sensory impairment (vs no sensory impairment), with a higher hazard of incident dementia; however, these estimates lacked precision. Hwang et al^23^ also reported that functional dual sensory impairment was associated with a 86% higher hazard of all-cause dementia over 8 years in a highly educated and mostly White sample of older adults. Associations were weaker among those reporting a single sensory impairment. Additionally, in the English Longitudinal Study of Aging,^31^ individuals with poor and moderate self-reported hearing were observed to be associated with having a 57% and 39% higher hazard of incident dementia over 9 years, respectively.

The observed associations between functional sensory impairments and dementia may be explained through several potential mechanisms. Sensory impairments may causally increase dementia risk through changes in brain structure and function, particularly through increases in cognitive load. In older adults with sensory impairment, greater cognitive resources are needed to support visual and auditory function, thus leaving fewer resources available to support cognitive tasks.^9,10^ Additionally, sensory impairment can lead to depression,^11,12^ social isolation,^13,14,15,16^ and reduced physical activity,^17,18,19^ all known factors associated with increased risk for dementia.^3^ The higher risk associated with dual sensory impairment, in particular, may also be because of the limited ability of individuals to compensate for single sensory impairment by employing functioning of an unimpaired sensory system. It is also possible that the observed associations are attributable to a “common cause,” such as microvascular changes and inflammation, that is responsible for both sensory impairment and dementia.^9^ Additional research is needed to test hypotheses regarding potential mechanisms.

### Implications

There are several important research and policy implications from our study. First, dual sensory impairment may be an important consideration in future studies of sensory impairment. Studies focusing only on single sensory impairment may miss an important component of the sensory impairment–dementia association given the observed, strong contribution of dual sensory impairment to dementia risk. Second, sensory impairments are potentially modifiable and may be a target for interventions aiming to reduce risk for dementia.^32^ Observational studies show improved cognition among older adults who receive cataract surgery to treat vision impairment.^33,34,35^ Additionally, the Aging and Cognitive Health Evaluation in Elders randomized control trial pilot study has shown the potential efficacy of hearing treatment in slowing memory decline; however, this was in a small pilot study, and more research is needed.^36^ No existing interventions for reducing dementia risk, to our knowledge, have provided treatment for both hearing and vision impairment in older adults with dual sensory impairment. Third, because of high prevalence and associations with poor health outcomes, sensory impairments are important considerations for public health and health care policy. Limited use of sensory aids among older adults can be attributed to Medicare’s restricted coverage of sensory aids.^37^ Revising Medicare policy to broaden access to sensory aids may significantly improve quality of life and reduce health care costs.

### Limitations

Our study has several limitations that should be considered. First, while dementia was not clinically assessed, the dementia classification used showed high sensitivity and specificity compared with clinically diagnosed dementia.^28^ Second, functional sensory impairment is a measure of perceived impairment, and participants may have under- or overreported their impairment compared with objectively measured impairment. We were also unable to investigate the role of assistive devices among participants with sensory impairment because the use of assistive devices was included in the definition of functional sensory impairment. Third, in cross-sectional analyses, participants’ report of functional sensory impairments may be affected by their cognitive status and thus observed cross-sectional associations between sensory impairments and dementia could have resulted from reverse causation. However, we did observe longitudinal associations between sensory impairment and increased dementia risk, suggesting that observed cross-sectional associations cannot be purely explained by reverse causation. Fourth, differential drop-out over time of participants who were more likely to be sensory impaired and at risk for dementia could have led to conservative bias (see eTable 3 in the Supplement). Our estimates of longitudinal dementia risk may have underestimated the true association; however, prevalence and cross-sectional estimates were nationally representative. Fifth, participants residing in nursing homes or other residential facilities were excluded from this analysis because dementia status and sensory impairment measures were not available for that portion of the sample. Thus, our results were generalizable to community-dwelling Medicare beneficiaries in the US.

## Conclusions

In this nationally representative sample of community-dwelling Medicare beneficiaries in the US, functional vision, hearing, and dual sensory impairment was common in older adults. Older adults with functional sensory impairments, particularly dual sensory impairment, were at increased risk for incident dementia. As the population of older adults with sensory impairment rises, further research is needed to identify prevention strategies to reduce the risk of dementia among older adults with sensory impairment.

## Notes

Supplement. **eFigure.** Kaplan-Meier Survival Curves for Adults by Functional Vision and Hearing Impairment Status: National Health and Aging Trends Study, 2011-2018 **eTable 1.** Discrete Time Survival Analysis Modeling the 2-Year Lagged Association Between Sensory Impairment and Incident Dementia **eTable 2.** Discrete Time Survival Analysis Modeling the Association Between Sensory Impairment and Incident Dementia Among Participants Aged 65 to 85 Years **eTable 3.** Distribution of Baseline Characteristics Among Participants Stratified by Censoring Status in Discrete Time Survival Analysis
