# Supplementary material for: Prevalence of Concurrent Functional Vision and Hearing Impairment and Association With Dementia in Community-Dwelling Medicare Beneficiaries
Source: JAMA Netw Open. 2021 Mar 19;4(3):e211558. doi: 10.1001/jamanetworkopen.2021.1558 (PMC8601132; doi:10.1001/jamanetworkopen.2021.1558)
Supplement: Supplement. — eFigure. Kaplan-Meier Survival Curves for Adults by Functional Vision and Hearing Impairment Status: National Health and Aging Trends Study, 2011-2018 eTable 1. Discrete Time Survival Analysis Modeling the 2-Year Lagged Association Between Sensory Impairment and Incident Dementia eTable 2. Discrete Time Survival Analysis Modeling the Association Between Sensory Impairment and Incident Dementia Among Participants Aged 65 to 85 Years eTable 3. Distribution of Baseline Characteristics Among Participants Stratified by Censoring Status in Discrete Time Survival Analysis [file jamanetwopen-e211558-s001.pdf]

Supplemental Online Content

Kuo PL, Huang AR, Ehrlich JR, et al. Prevalence of concurrent functional vision and hearing impairment and association with dementia in community-dwelling Medicare beneficiaries. *JAMA Netw Open*. 2021;4(3):e211558. doi:10.1001/jamanetworkopen.2021.1558

eFigure. Kaplan-Meier Survival Curves for Adults by Functional Vision and Hearing Impairment Status: National Health and Aging Trends Study, 2011-2018

eTable 1. Discrete Time Survival Analysis Modeling the 2-Year Lagged Association Between Sensory Impairment and Incident Dementia

eTable 2. Discrete Time Survival Analysis Modeling the Association Between Sensory Impairment and Incident Dementia Among Participants Aged 65 to 85 Years

eTable 3. Distribution of Baseline Characteristics Among Participants Stratified by Censoring Status in Discrete Time Survival Analysis

This supplemental material has been provided by the authors to give readers additional information about their work.

© 2021 Kuo PL et al. *JAMA Network Open.*

eFigure. A. Kaplan-Meier survival curves for adults 65 years and older by functional vision and hearing impairment status: National Health and Aging Trends Study, 2011-2018

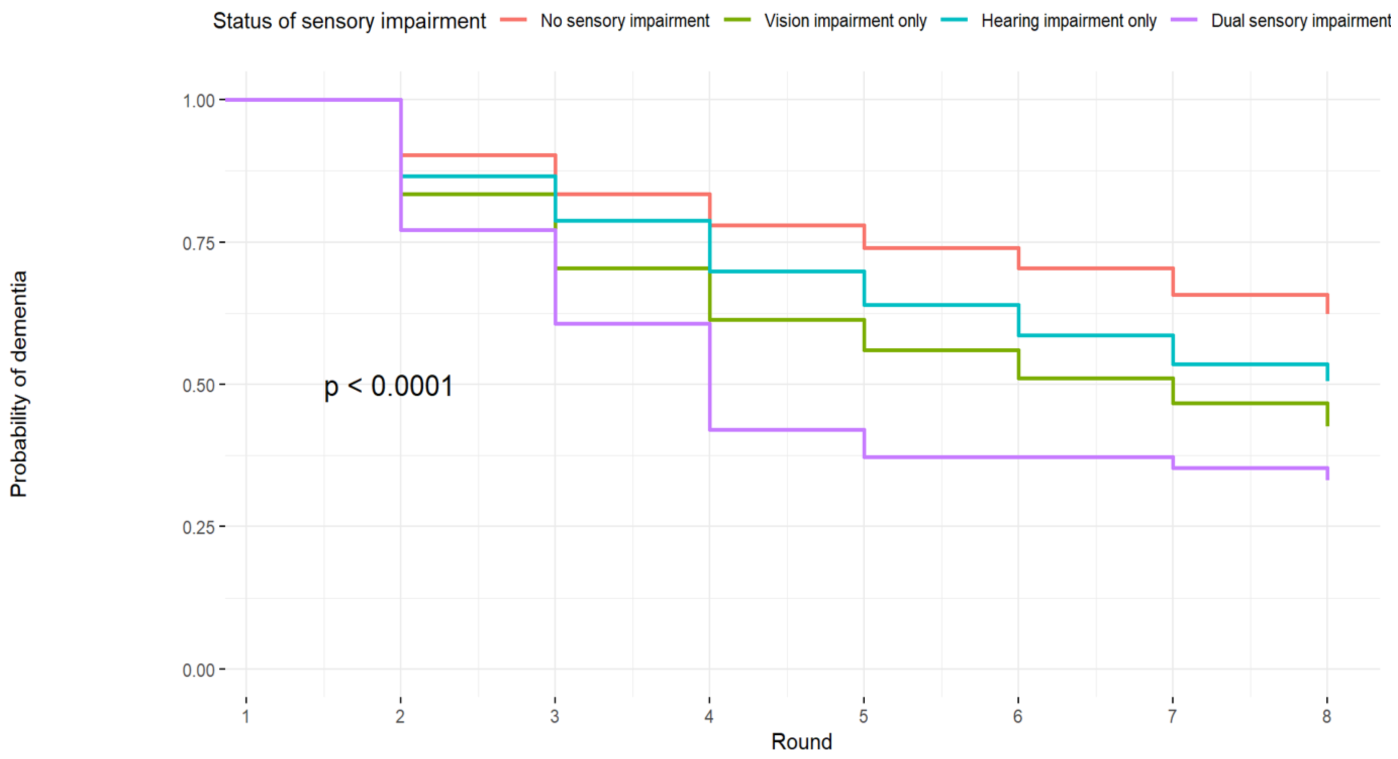

Number at Risk

No sensory impairment	3357	3357	2538	2037	1804	1601	1402	1227
Vision impairment only	200	200	141	101	81	68	58	46
Hearing impairment only	878	878	669	519	429	358	292	249
Dual sensory impairment	96	96	61	39	27	21	19	16

Cumulative Number of Events

No sensory impairment	0	326	519	651	744	820	914	978
Vision impairment only	0	33	55	68	75	81	86	90
Hearing impairment only	0	118	178	237	273	303	328	342
Dual sensory impairment	0	22	35	47	50	50	51	52

© 2021 Kuo PL et al. *JAMA Network Open.*

B. Kaplan-Meier survival curves for adults 85 years and older by functional vision and hearing impairment status: National Health and Aging Trends Study, 2011-2018

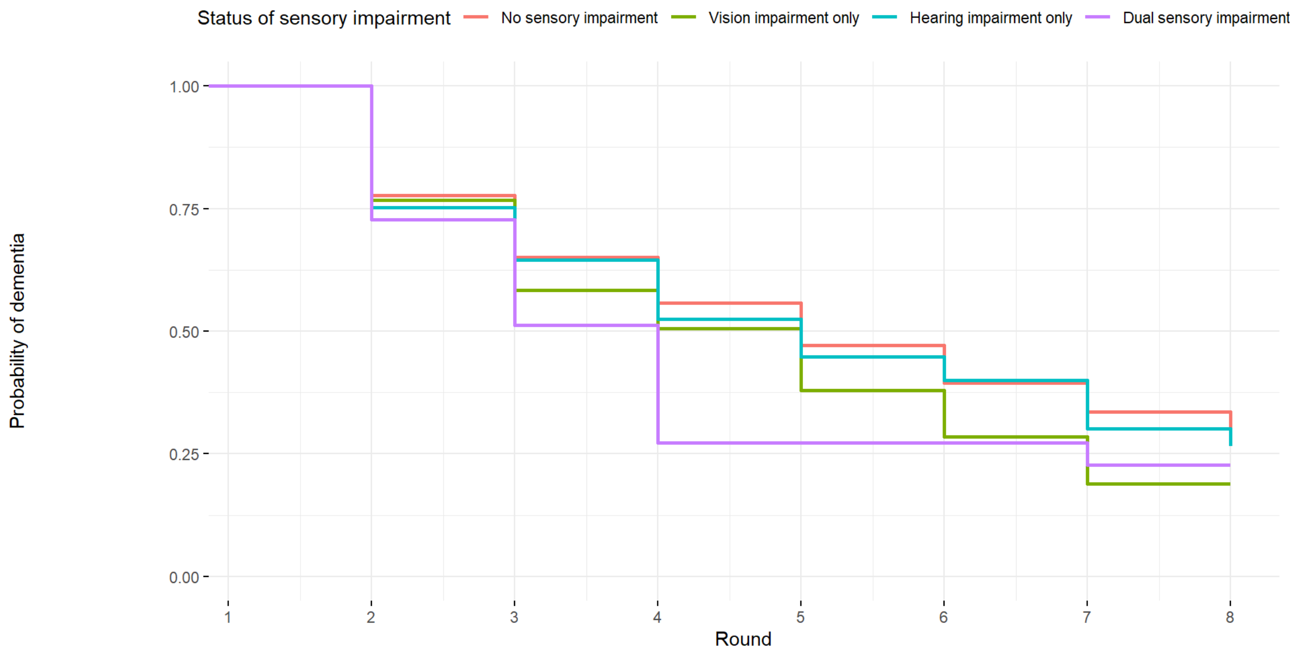

Number at Risk

	1	2	3	4	5	6	7	8
No sensory impairment	318	318	204	147	116	86	60	43
Vision impairment only	43	43	25	15	12	8	6	3
Hearing impairment only	250	250	163	117	83	64	49	34
Dual sensory impairment	44	44	27	15	8	7	6	3

Cumulative Number of Events

	1	2	3	4	5	6	7	8
No sensory impairment	0	71	104	125	143	157	166	173
Vision impairment only	0	10	16	18	21	23	25	25
Hearing impairment only	0	62	85	107	119	126	138	142
Dual sensory impairment	0	12	20	27	27	27	28	28

© 2021 Kuo PL et al. *JAMA Network Open.*

C. Kaplan-Meier survival curves for older adults 65-84 years by functional vision and hearing impairment status: National Health and Aging Trends Study, 2011-2018

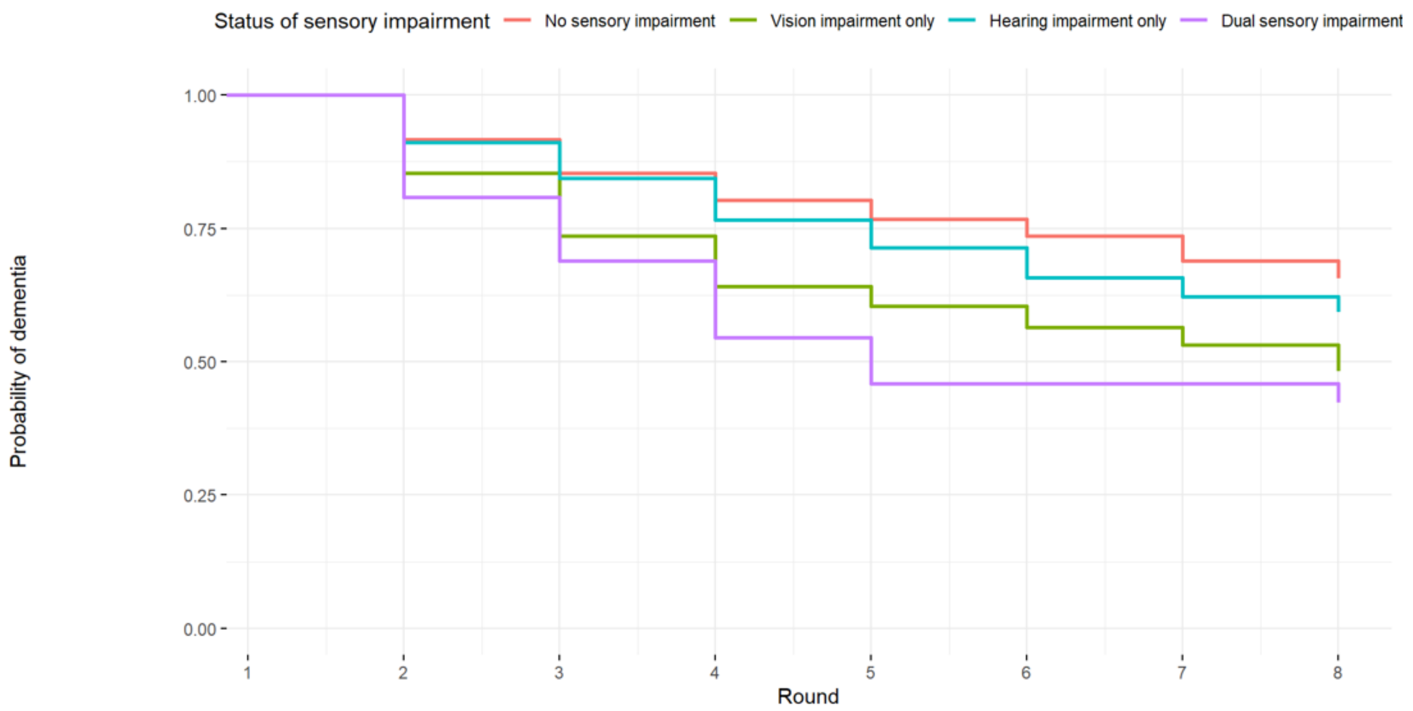

Number at Risk

No sensory impairment	3039	3039	2334	1890	1688	1515	1342	1184
Vision impairment only	157	157	116	86	69	60	52	43
Hearing impairment only	628	628	506	402	346	294	243	215
Dual sensory impairment	52	52	34	24	19	14	13	13

Cumulative Number of Events

No sensory impairment	0	255	415	526	601	663	748	805
Vision impairment only	0	23	39	50	54	58	61	65
Hearing impairment only	0	56	93	130	154	177	190	200
Dual sensory impairment	0	10	15	20	23	23	23	24

© 2021 Kuo PL et al. *JAMA Network Open.*

eTable 1. Discrete Time Survival Analysis Modeling the 2-Year Lagged Association Between Sensory Impairment and Incident Dementia[a,b]

	Model 1		Model 2		Model 3	
Variables	Hazard Ratio (95%CI)		Hazard Ratio (95%CI)		Hazard Ratio (95%CI)	
Status of sensory impairment						
No sensory impairment	1 (Reference)		1 (Reference)		1 (Reference)	
Vision impairment only	1.77	1.25 – 2.49	1.42	1.01 – 2.02	1.42	1.00 – 2.01
Hearing impairment only	1.58	1.32 – 1.89	1.30	1.07 – 1.57	1.29	1.07 – 1.56
Dual sensory impairment	4.11	2.44 – 6.92	2.20	1.28 – 3.76	2.15	1.25 – 3.69

Model 1 is the unadjusted model.
In Model 2, age, sex, levels of education, and race/ethnicity were included.
In Model 3, age, sex, levels of education, race/ethnicity, smoking, hypertension, diabetes, stroke, heart attack, heart disease, lung disease, and cancer were included.
[a] Assessment of dementia risk began 2 years after the measurement of sensory impairment. Participants who developed dementia within two years of measurement of sensory impairment were excluded
[b] The proportional hazard assumption was checked, and time-varying hazard ratios were allowed when the assumption was violated. The proportional hazard assumption held for the functional hearing impairment group (vs. no impairment) and for the functional vision impairment group (vs. no impairment) across the 7-year follow-up period. For the functional dual sensory impairment group (vs. no impairment), the proportional hazard assumption held across the first 4-year follow-up period but did not hold after the 4-year follow-up. Time-varying hazard ratios were allowed between the dual sensory impairment group (vs. no impairment) after 4-year follow-up.

© 2021 Kuo PL et al. *JAMA Network Open.*

eTable 2. Discrete Time Survival Analysis Modeling the Association Between Sensory Impairment and Incident Dementia Among Participants Aged 65 to 85 Years [a]

	Model 1		Model 2		Model 3	
Variables	Hazard Ratio (95%CI)		Hazard Ratio (95%CI)		Hazard Ratio (95%CI)	
Status of sensory impairment						
No sensory impairment	1 (Reference)		1 (Reference)		1 (Reference)	
Vision impairment only	1.80	1.40 – 2.32	1.55	1.20 – 2.00	1.47	1.14 – 1.91
Hearing impairment only	1.23	1.05 – 1.43	1.08	0.92 – 1.27	1.04	0.89 – 1.23
Dual sensory impairment	2.74	1.81 – 4.16	1.69	1.10 – 2.58	1.65	1.07 – 2.52

Model 1 is the unadjusted model.
In Model 2, age, sex, levels of education, and race/ethnicity were included.
In Model 3, age, sex, levels of education, race/ethnicity, smoking, hypertension, diabetes, stroke, heart attack, heart disease, lung disease, and cancer were included.
[a] The proportional hazard assumption was checked, and time-varying hazard ratios were allowed when the assumption was violated. The proportional hazard assumption held for the functional hearing impairment group (vs. no impairment) and for the functional vision impairment group (vs. no impairment) across the 7-year follow-up period. For the functional dual sensory impairment group (vs. no impairment), the proportional hazard assumption held across the first 4-year follow-up period but did not hold after the 4-year follow-up. Time-varying hazard ratios were allowed between the dual sensory impairment group (vs. no impairment) after 4-year follow-up.

© 2021 Kuo PL et al. *JAMA Network Open.*

eTable 3. Distribution of Baseline Characteristics Among Participants Stratified by Censoring Status in Discrete Time Survival Analysis

	Censored At End of Study	Censored Prior to End of Study	Developed Dementia
n	1455	1614	1462
Age group			
65-69	475 (32.6)	390 (24.2)	170 (11.6)
70-74	408 (28.0)	384 (23.8)	272 (18.6)
75-79	310 (21.3)	332 (20.6)	303 (20.7)
80-84	190 (13.1)	293 (18.2)	349 (23.9)
85-89	58 (4.0)	141 (8.7)	232 (15.9)
90 +	14 (1.0)	74 (4.6)	136 (9.3)
Sensory impairment			
No Impairment	1163 (79.9)	1216 (75.3)	978 (66.9)
Vision Impairment only	42 (2.9)	68 (4.2)	90 (6.2)
Hearing Impairment only	235 (16.2)	301 (18.6)	342 (23.4)
Dual Sensory Impairment	15 (1.0)	29 (1.8)	52 (3.6)
Education			
Less than high school	167 (11.5)	290 (18.0)	439 (30.0)
High school	358 (24.6)	499 (30.9)	403 (27.6)
More than high school	930 (63.9)	825 (51.1)	620 (42.4)
Male Sex	594 (40.8)	707 (43.8)	592 (40.5)
Race			
Non-Hispanic White	1201 (82.5)	1170 (72.5)	962 (65.8)
Hispanic	41 (2.8)	63 (3.9)	106 (7.3)
Non-Hispanic Black	190 (13.1)	331 (20.5)	355 (24.3)
Others	23 (1.6)	50 (3.1)	39 (2.7)
Hypertension	901 (61.9)	1116 (69.1)	1020 (69.8)
Diabetes	275 (18.9)	386 (23.9)	418 (28.6)
Stroke	85 (5.8)	149 (9.2)	174 (11.9)
Heart Attack	131 (9.0)	244 (15.1)	243 (16.6)

© 2021 Kuo PL et al. *JAMA Network Open.*

Heart Disease	211 (14.5)	307 (19.0)	259 (17.7)
Lung Disease	202 (13.9)	258 (16.0)	211 (14.4)
Cancer	360 (24.7)	452 (28.0)	385 (26.3)
Ever smoked	733 (50.4)	698 (43.2)	762 (52.1)

© 2021 Kuo PL et al. *JAMA Network Open.*
